# Randomized trial protocol of interscalene nerve block vs liposomal bupivacaine injection after total shoulder arthroplasty

**DOI:** 10.1097/MD.0000000000020968

**Published:** 2020-07-10

**Authors:** Jianbin He, Yalan Li

**Affiliations:** a Department of Anesthesiology, The Second Clinical Medical College, Jinan University/Shenzhen People's Hospital; b Department of Anesthesiology, The first affiliated hospital of Jinan University, Guangdong Province, China.

**Keywords:** interscalene blockade, local infiltration analgesia, random, study protocol, total shoulder arthroplasty

## Abstract

**Background:**

The possibility of local infiltration analgesia (LIA) replacing interscalene blockade (ISB) as an integral component of a multimodal clinical pathway for total shoulder arthroplasty (TSA) needs to be further investigated. We thus further designed a randomized controlled study to compare LIA with ISB in the treatment of TSA.

**Methods:**

This blinded and randomised study was performed after approval of the institutional review board in the first affiliated hospital of Jinan University. The included patients were all aged over 18 years and underwent shoulder arthroplasty because of osteoarthritis of the shoulder. Subjects were randomized into 2 groups as follows: LIA or ISB. The primary outcome of this noninferiority study is opioid consumption within the first 24 hours following surgery. Secondary outcomes included pain scores, length of hospital stay, complication, and satisfaction score. *P* value < .05 was considered statistically significant.

**Results:**

For the present trial, we hypothesized that there would be no difference in pain score levels and opioid medication use throughout admission.

**Trial registration:**

This study protocol was registered in Research Registry (researchregistry5640).

## 1 Introduction

Currently, total shoulder arthroplasty (TSA) has been widely used in treatment for patients with degenerative arthritis and rotator-cuff-deficient conditions of the glenohumeral joint.^[1,2]^ The annual number of TSA is rising with the growing elderly population. The growth rates of TSA are higher than the rates for total hip and knee procedures in the United States and were predicted to further increase by between 192% and 322% by 2015 based on 2008 numbers.^[3]^ However, due to the soft tissue injury and large amount of bone destruction involved, undesirable postoperative pain remains a challenge for both patients and surgeons after TSA.

Pain management after TSA is an important variable in the perioperative period that can influence participation in physical therapy, discharge from the hospital or outpatient surgery center, and patient satisfaction. In addition, inadequate pain management has been shown to contribute to an increased incidence of postoperative complications.^[4]^ In shoulder literature, various techniques can be used to relieve postoperative pain, including anti-inflammatory medications, opioids, epidural anesthesia, interscalene blockade (ISB), and local infiltration analgesia (LIA). The ISB allows delivery of local anesthetic in a controlled manner to the trunks of the brachial plexus between the anterior and middle scalene muscles.^[5]^ Compared with patients receiving only general anesthesia, patients who undergo preoperative ISB have shorter hospital stays and a reduced need for analgesics.^[6]^ However, ISB is also associated with risks, such as failure of nerve blockade, residual neurapraxia, displacement of the catheter postoperatively, systemic toxicity, and respiratory and neurologic complications.^[7]^ LIA has recently gained popularity for its potential to provide extended postoperative pain relief. It is a surgeon-controlled analgesic technique that used to reduce the pain in the early postoperative period with no influence on muscle strength. However, the possibility of LIA replacing ISB as an integral component of a multimodal clinical pathway for TSA needs to be further investigated.

Despite the evidence above, concerns exist regarding the incomplete analgesia of LIA. Previous studies have been published to explore the efficacy between LIA vs ISB in reducing pain after TSA, but with different conclusions.^[4,6,8]^ We thus further designed a randomised controlled study to compare LIA with ISB in the treatment of TSA. For the present trial, we hypothesized that there would be no difference in pain score levels and opioid medication use throughout admission.

## 2 Material and method

**Figure 1:**
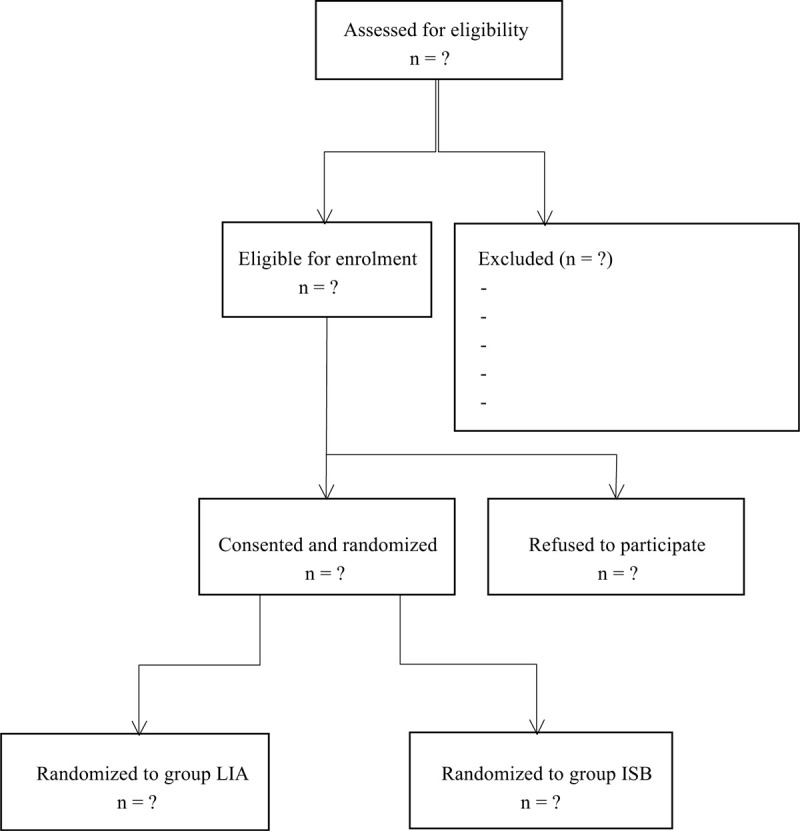
Consolidated Standards of Reporting Trials Statement flow diagram.

This blinded and randomised study was performed after approval of the institutional review board in the first affiliated hospital of Jinan University (GNJN011258). It was carried out in accordance with the principles of the Helsinki Declaration. Data are presented according to the CONSORT statement. This trial was also registered in Research Registry (researchregistry5640). The flowchart of this trial is shown in Figure 1.

### 2.1 Study participants

The included patients were all aged over 18 years and underwent shoulder arthroplasty because of osteoarthritis of the shoulder. The exclusion criteria were as follows: contraindication to ISB (respiratory failure or peripheral oxygen saturation <90% in ambient air), history of allergy to one of the treatments in the study, chronic opioid treatment for more than 3 months, history of shoulder surgery on the same side, weight of less than 50 kg, American Society of Anesthesiologists score greater than 3, and/or incapacity to provide informed consent and pain self-assessment.

### 2.2 Randomization and blinding

After written informed consent was taken, patients were randomly allocated to one of the 2 groups in a 1:1 ratio using a computer-generated list of random numbers with a block randomization technique (www.randomization.com) by a research assistant. A unique randomization code was used with no restrictions to either of the 2 study groups: LIA or ISB. The results of the randomization were maintained in opaque envelopes and stored with the research coordinator. The patient, anesthesiologist, surgeon, physiotherapists, acute pain nurses, and outcome assessors were unaware of study group allocation.

### 2.3 Intervention

#### 2.3.1 LIA group

Patients allocated to the LIA treatment group received local infiltration of 20 ml of LB (266 mg) mixed in 20 ml of sterile saline. This mixture was infiltrated locally using a standardized protocol at the completion of component implantation and before closure of the wound. The injection protocol was as follows. A 60-ml syringe with a 1-inch, 18-gauge needle was used to administer the injection; 5 ml was injected into the periosteum; 10 ml was injected into the deltoid in 2-ml increments spread over the deltoid muscle anteriorly; 10 ml was injected into the pectoralis muscle, again in 2-ml increments; and the final 15 ml was injected evenly along the incision before wound closure. After surgery, patients in both groups were admitted to the orthopedic floor. They were prescribed a standardized postoperative pain regimen consisting of 650 mg acetaminophen every 8 hours, oxycodone 5 mg every 4 hours as needed for pain levels <5, oxycodone 10 mg every 4 hours as needed for pain levels >5, and morphine 2 mg intravenously every 4 hours as needed for severe breakthrough pain.

#### 2.3.2 ISB group

A perineural interscalene catheter was inserted before surgery under ultrasound control by a senior anesthesiologist. The anesthesiologist injected 20 ml of 0.2% ropivacaine during the TSA procedure after a negative aspiration test to confirm the absence of blood reflux in the catheter. In the recovery room, a continuous perfusion of 0.2% ropivacaine was started at 5 ml/hour for 48 hours; then, the catheter was removed.

### 2.4 Outcome measures

The primary outcome of this noninferiority study is opioid consumption within the first 24 hours following surgery. Subjects receive standardized postoperative multimodal analgesics. Supplemental oxycodone 5 to 15 mg every 3 hours for pain is available to each subject. Nurses administer a 5-mg oxycodone tablet for numerical rating scale (NRS) scores of 1 to 3, 10 mg for NRS scores of 4 to 6, and 15 mg for NRS scores of 7 to 10. Oral hydromorphone is substituted for oxycodone in the instances of patient allergy or intolerance. Rescue analgesia is available with IV hydromorphone 0.5 mg every 1 hour as needed for pain of greater than NRS of 7 when refractory to oral opioids. Opioid consumption during the first 48 hours postoperatively is retrieved from the electronic medical record and convert to IV morphine equivalents for analysis. The average and worst NRS pain scores at rest and with activity are determined by blinded investigators at 24 and 48 hours postoperatively.

Secondary outcomes included pain scores, length of hospital stay, complication, and satisfaction score. Pain scores were recorded at 1, 6, 12, 24, and 48 hours post-operatively, using a NRS (0–10) at rest and during 45-degree passive flexion of shoulder. Length of hospital stay was calculated by measuring the time from the completion of surgery through discharge for each patient.

### 2.5 Statistical analysis

All the data were analyzed using SPSS v. 24 (IBM Corp., Armonk, NY, USA). We used the Kolmogorov–Smirnov test to assess whether variable distributions violated the assumption of normality. Data are presented as mean and standard deviation or with medians and 25th to 75th percentiles as appropriate. The normal distributed numerical variable (NRS scores, opioid consumption, length of hospital stay, and patient satisfaction scores) was analyzed by Student *t* test. If the numerical variable has a non-normal distribution or unequal variance, the Wilcoxon Mann–Whitney *U* test was used (ASA grade); Pearson Chi-Squared test or Fisher exact test was used to analyze the qualitative variable (complication). The nature of the hypothesis testing was 2-tailed, and a *P* value <.05 was considered statistically significant.

## 3 Discussion

Regional anesthesia in the form of ISB has proved to be an effective mode of postoperative analgesic control for patients undergoing TSA. The safety and complication rates of INB have been evaluated in various studies.^[9–12]^ Weber and Jain evaluated the efficacy of ISB in a review of 218 patients. They found that 13% of ISBs in their study failed, and 5% of their patients had an abnormal neurologic response the day after surgery. Misamore et al demonstrated that 16% of patients undergoing ISB experience immediate postoperative block side effects, with 4.4% of patients experiencing persistent neurologic complications.^[13]^ This study displayed similar results, with 3% of patients experiencing a persistent neurologic complication. A study by Fredrickson and Price suggested that an increase in postoperative motor blockade can be experienced with ISB and is associated with a reduction in patient satisfaction.^[14]^ These findings along with the desire for early mobilization of the operative extremity suggest the utility of an alternative method for pain control in shoulder arthroplasty.

The purpose of this study was to compare the efficacy of LIA and ISB in immediate postoperative pain control as measured by patients subjective pain scores and in-hospital opioid medication use. We hypothesized that there would be no difference in pain score levels and opioid medication use throughout admission. The main limitation is that the clinical effects and complications of ISB were compared with that of LIA without the control group in the present study. According to the beneficence principle for patients, no control group with placebo was designed in the protocol of the present study. We do not think this limitation would affect the results tremendously.

## Author contributions

**Conceptualization:** Jianbin He.

**Data curation:** Jianbin He.

**Formal analysis:** Jianbin He.

**Funding acquisition:** Yalan Li.

**Investigation:** Jianbin He, Yalan Li.

**Methodology:** Jianbin He, Yalan Li.

**Resources:** Yalan Li.

**Software:** Yalan Li.

**Supervision:** Yalan Li.

**Validation:** Jianbin He.

**Visualization:** Jianbin He.

**Writing – original draft:** Jianbin He, Yalan Li

**Writing – review & editing:** Yalan Li.
